# p600 Plays Essential Roles in Fetal Development

**DOI:** 10.1371/journal.pone.0066269

**Published:** 2013-06-18

**Authors:** Takeo Nakaya, Kei-ichiro Ishiguro, Camille Belzil, Anna M. Rietsch, Qunyan Yu, Shin-ichi Mizuno, Roderick T. Bronson, Yan Geng, Minh Dang Nguyen, Koichi Akashi, Piotr Sicinski, Yoshihiro Nakatani

**Affiliations:** 1 Department of Cancer Biology, Dana-Farber Cancer Institute and Harvard Medical School, Boston, Massachusetts, United States of America; 2 Translational Research Unit and Department of Molecular Pathology, Tokyo Medical University, Shinjuku, Tokyo, Japan; 3 Institute of Molecular and Cellular Biosciences, University of Tokyo, Bunkyo, Tokyo, Japan; 4 Hotchkiss Brain Institute, University of Calgary, Calgary, Alberta, Canada; 5 Department of Cancer Immunology and AIDS, Dana-Farber Cancer Institute and Harvard Medical School, Boston, Massachusetts, United States of America; 6 Department of Medicine and Biosystemic Science, Kyushu University Graduate School of Medical Science, Fukuoka, Japan; 7 Dana-Farber/Harvard Cancer Center, Harvard Medical School, Boston, Massachusetts, United States of America; Cincinnati Children’s Hospital Medical Center, United States of America

## Abstract

p600 is a multifunctional protein implicated in cytoskeletal organization, integrin-mediated survival signaling, calcium-calmodulin signaling and the N-end rule pathway of ubiquitin-proteasome-mediated proteolysis. While *push*, the *Drosophila* counterpart of *p600*, is dispensable for development up to adult stage, the role of *p600* has not been studied during mouse development. Here we generated *p600* knockout mice to investigate the in vivo functions of p600. Interestingly, we found that homozygous deletion of *p600* results in lethality between embryonic days 11.5 and 13.5 with severe defects in both embryo and placenta. Since *p600* is required for placental development, we performed conditional disruption of *p600*, which deletes selectively *p600* in the embryo but not in the placenta. The conditional mutant embryos survive longer than knockout embryos but ultimately die before embryonic day 14.5. The mutant embryos display severe cardiac problems characterized by ventricular septal defects and thin ventricular walls. These anomalies are associated with reduced activation of FAK and decreased expression of MEF2, which is regulated by FAK and plays a crucial role in cardiac development. Moreover, we observed pleiotropic defects in the liver and brain. In sum, our study sheds light on the essential roles of p600 in fetal development.

## Introduction

p600, also known as UBR4, is a 600 kDa cellular protein that is ubiquitously expressed and plays various roles depending on cell type [1], [2]. One obvious structural motif of p600 is the UBR box of the N-end rule pathway ubiquitin ligases. The UBR box is responsible for recognition of the N-terminus of their substrate proteins that are produced by protein processing and/or modifications in response to environmental conditions [3]–[6]. Additionally, p600 has a non-canonical calmodulin-binding domain and binds to calmodulin in a calcium-dependent manner [1]. Moreover, p600 associates with the cytoskeleton to impact cell morphology and intracellular transport [1], [7].

Regarding its biological functions, p600 is involved in activation of integrin-mediated survival signaling pathways in adherent cells. Suppression of p600 expression by short hairpin RNA (shRNA) abrogates formation of integrin-mediated ruffled membranes and cellular polarity. These phenotypes in the knockdown cells are associated with reduced activation of focal adhesion kinase (FAK), which plays a role in integrin-mediated survival signaling pathways [1]. p600 functions not only in anchorage-dependent growth but also in anchorage-independent growth. Suppression of p600 expression prevents anchorage-dependent growth in various cancer cells including osteocarcinoma, cervical cancers, and gastric cancers [8]–[10]. Moreover, knockdown of p600 suppresses growth of gastric cancer cells in SCID mice [10]. Although the exact molecular mechanisms whereby p600 contributes to cancer growth still remain unclear, p600 has been shown to be a direct target for viral oncoproteins, namely, human and bovine papillomavirus E7 [8], [9], [11]. Importantly, experiments with papillomavirus E7 mutants demonstrated a relation between p600-binding activity and transforming activity [8], [9]. Moreover, suppression of either E7 or p600 leads to loss of ability in anchorage-independent growth [8], [9], [12]. Thus, formation of the E7-p600 complex in transformed cells could be crucial for anchorage-independent growth presumably by inhibiting apoptosis. Likewise, association of p600 with cellular factors may be disorganized in nonvirus-mediated cancers, although such factors have not been identified yet.

p600 functions not only in proliferating cells but also in non-dividing cells. p600 plays an essential role in neurite outgrowth and neuronal migration during brain development through regulation of microtubule stability and transport of endoplasmic reticulum transport [7]. Neurological functions are also reported for the *Drosophila* counterpart of *p600*, termed *push*. Mutation in *push* does not cause developmental effects but triggers defects in synaptic transmission at neuromuscular junctions in adult flies [13].

In *Arabidopsis*, the p600 counterpart BIG plays a role in polar-dependent intercellular transport of auxin [14], a plant hormone essential for development. Specific distribution of auxin via polar transport is crucial for the regulation of auxin action and thus mutations in *BIG* cause developmental defects. As a mechanism, polar localization of auxin efflux carrier PIN proteins to a particular face of the plasma membrane enables the polar auxin transport [15], [16]. PIN proteins dynamically cycle between the plasma membrane and endosomes. Auxin prevents the internalization step of PIN proteins in a BIG-dependent manner, resulting accumulation of PIN proteins at the plasma membrane [17]. Despite the roles assigned to p600’s counterparts, the physiological functions of p600 in mammal remain poorly understood. To gain insights into the in vivo roles of p600 we generated *p600* knockout mice. Here we demonstrate that the embryonic p600 plays essential roles during fetal development.

## Materials and Methods

### Generation of p600 Deficient Mice

**Figure 1:**
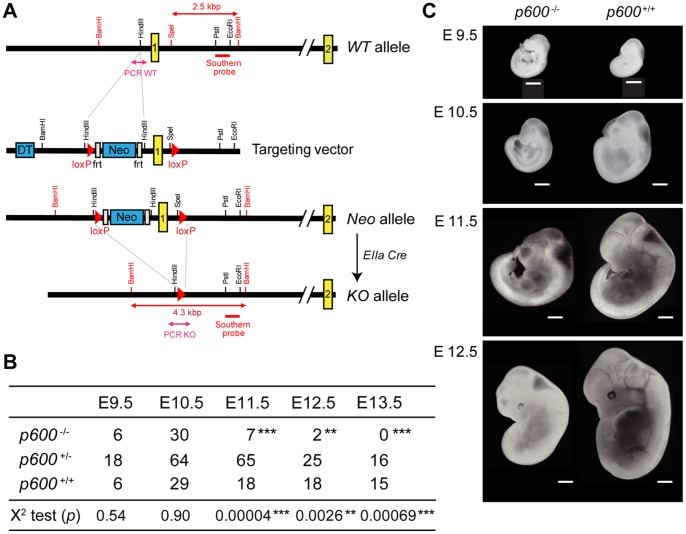
Disruption of *p600* results in growth retardation and lethality during embryonic development. (A) Targeting strategy of *p600* locus. The exon encoding the initiating methionine codon of *p600* allele was replaced with a Neo cassette by homologous recombination in ES cell lines. The ES cell lines were microinjected into mouse blastocysts to generate chimeric mice. These chimeras were bred to obtain offspring that are heterozygous for ‘Neo allele’. *p600* ^Neo/+^ mice were then crossed with *EIIa-Cre* transgenic mouse [19] to delete the loxP flanked region, yielding ‘KO allele’. The locations of PCR amplicons used for genotyping are as indicated. Restriction enzyme digestion of genomic DNA with BamHI and SpeI produces the 2.5 and 4.3 kbp fragments from WT and Neo alleles, respectively, that hybridize with the probe for Southern blotting. The regions for PCR genotypings are indicated. (B) The genotypes of living embryos produced by inter-crossing of *p600* ^+/−^ mice. The numbers of embryos with indicated genotypes from days E9.5 to E13.5 are shown. Significances of the frequency of survival *p600* ^−/−^ embryos and expected frequency of 25%, according to Mendelian distribution of the genotypes were calculated by X^2^ test. The *p*-values of X^2^ test are shown. ** and *** show p-value <0.01 and <0.001, respectively. (C) The appearances of typical embryos of *p600* ^−/−^ and *p600* ^+/+^ littermates from days E9.5 to E12.5. Scale bars indicate 1 mm.

Isolation of recombinant ES cell clones and blastocysts injection were performed at The Dana-Farber Transgenic Core Facility. Briefly, ES cells were electroporated with the purified NotI-linearlized targeting vector constructed from pKOII [18] (see Figure 1A). Germline transmission was obtained by mating male chimeras with C57BL/6 females.

*EIIa* promoter-driven *Cre* transgenic mice [B6.FVB-Tg(EIIa-cre)C5379Lmgd/J], *Sox2* promoter-driven *Cre* transgenic mice [STOCK Tg(Sox2-cre)1Amc/J], and *ß-actin* promoter-driven flippase transgenic mice [B6;SJL-Tg(ACTFLPe)9205Dym/J] were purchased from Jackson Laboratory. Backcross was done with C57BL/6 stain and all the straight and conditional knockout mice were congenic with the C57BL/6 genetic background.

### Ethics Statement

All animal studies were approved by the Dana-Farber Institutional Animal Care and Use Committee (IACUC) (Animal Protocol Numbers 03-038 and 05-003) and were performed in strict accordance with IACUC guidelines.

### Southern Blot Analysis for Genotyping

Genomic DNA isolated from embryos were digested with BamHI and SpeI (Figure 1A), fractionated by agarose gel electrophoresis, and transferred onto nitrocellulose membrane. The membrane was hybridized with the ^32^P-dCTP-labeled 880 bp PstI and EcoRI fragment purified from the targeting plasmid (Figure 1A).

### PCR Analysis for Genotyping

PCR-based genotyping analysis was performed using genomic DNA isolated from embryos. The following primers were employed for PCR amplification. For detection of ‘WT allele’ (amplicon length of 158 bp), a set of primers, 5′-TGCTGCGATGGCTACTAATG-3′ and 5′-CCTATTTCGCAGCCACTTCTTT-3′, was used. For detection of ‘KO allele’ (amplicon length of 242 bp), a set of primers, 5′-AAAGACTGCTGCGATGGCTAC-3′ and 5′-GAGAAGGACTCCGATCTCGG-3′, was used. In the conditional knockout experiments, for detection of ‘floxed allele’ (amplicon length of 269 bp) and ‘WT allele’ (amplicon length of 235 bp), a set of primers, 5′-CACCAGAACCGGAGGTTTAC-3′ and 5′-CAGCAAGCCAAGGAACAGTC-3′, was used. For detection of *Sox2-Cre* transgene (amplicon length of 102 bp), a set of primers, 5′-GCGGTCTGGCAGTAAAAACTATC-3′ and 5′-GTGAAACAGCATTGCTGTCACTT-3′, was used. As an internal control for PCR reactions, interleukin-2 gene was coamplified (amplicon length of 324 bp) with a set of primers, 5′-CTAGGCCACAGAATTGAAAGATCT-3′ and 5′-GTAGGTGGAAATTCTAGCATCATCC-3′.

### Western Blot Analysis

For Western blot analysis, protein lysates were prepared from embryos by extracting with RIPA buffer. 10 µg of lysates were fractionated by SDS-PAGE and transferred to a Nitrocellulose membrane. The membranes were probed with anti-p600 [1] and -actin (I-19; Santa Cruz Biotechnology) antibodies and detected with horseradish peroxidase–conjugated secondary antibodies using ECL detection system.

### Histology and Immunohistochemistry

Tissues were fixed in Bouin’s solution or 4% paraformaldehyde in 0.1 M PBS for 24 hr, passed through graded alcohols, and embedded in paraffin. After deparaffinization, some sections were stained with Hematoxylin & Eosin (H&E). The surface areas were calculated using WinROOF software (Mitani Corporation).

For immunohistochemistry, paraformaldehyde-fixed samples were incubated with the primary antibodies after deparaffinization and heat-induced antigen retrieval. For fluorescent immunohistochemistry, samples were incubated with secondary antibody conjugated with Alexa Fluor 568 (Life Technologies). For colorimetric immunohistochemistry, samples were incubated with secondary antibody conjugated with polymeric horseradish peroxidase (Vector Laboratories) and visualized with 3,3′ Diaminobenzidine (DAB) chromogen (Dako). The antibodies employed were as follows: rabbit anti-laminin antibody (L9393, Sigma-Aldrich), rabbit anti-Ki-67 (VP-RM04, Vector Laboratories), rabbit anti-desmin (#5332, Cell Signaling Technology), FAK (#3285, Cell Signaling Technology), mouse anti-phospho-FAK (Tyr397) (#611806, BD Transduction Laboratories), mouse anti-MEF2 (sc-17785, Santa Cruz Biotechnology), and rabbit anti-cleaved caspase-3 (Asp175) (#9664, Cell Signaling Technology). TUNEL assays were performed by using ApoTag Peroxidase In Situ Apoptosis Detection Kit (EMD Millipore) according to the manufacturer’s protocol.

## Results

### *p600* is Required for Embryonic Development

To investigate the role of *p600* in mice, we generated floxed mutant mice, which possess loxP sites flanking the exon encoding the initiating methionine (Figure 1A). To delete the floxed alleles the mutant mice was crossed with *EIIa-Cre* transgenic mice, which ubiquitously express Cre recombinase at the zygote stage [19]. After two crosses we obtained homozygous *p600* knockout (KO) animals. Genotype analysis of the knockout embryos revealed that *p600* plays essential roles in embryonic development (Figures 1B and 1C, and S1). At embryonic day (E) 9.5, *p600* KO embryos developed in a population with Mendelian inheritance. Moreover, appearances and sizes of *p600* KO embryos were similar to those of wild type or heterozygous littermates (Figure 1B). However, at E10.5, a large proportion of living *p600* KO embryos were clearly smaller in size than wild type or heterozygous littermates. After E11.5, the total proportion of surviving *p600* KO embryos was significantly less than the predicted Mendelian ratio. This indicates that some of the knockout embryos start to die around E11.5 (Figure 1C). No living *p600* KO embryos were detected at E13.5, indicating that *p600* KO embryos die between E11.5 and E13.5 (Figure 1B). These results indicate that *p600* is essential for embryonic development.

### *p600* Knockout Impairs Placental Development with Thin Labyrinth Layer

**Figure 2:**
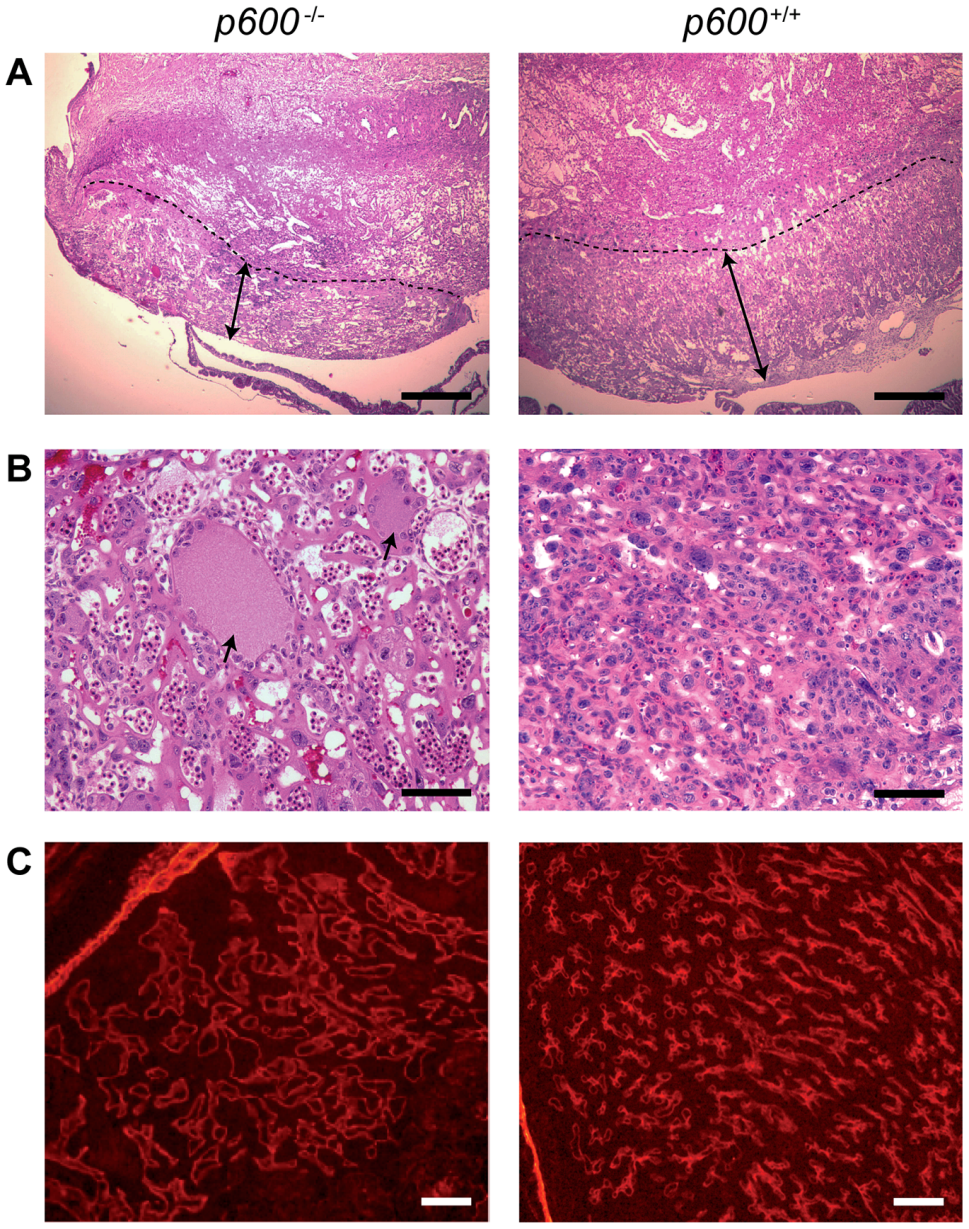
Placental abnormalities in *p600* ^−/−^ animals. (A) Placental labyrinth defects in *p600* ^−/−^ animals. H&E staining images of the labyrinth layer of *p600* knockout and control littermate at E12.5 (the area below the dashed lines) are shown. The labyrinth areas of *p600* ^−/−^ placenta are thinner than those of wild type littermate as shown by arrows. Scale bars indicate 500 µm. (B) High magnification images of the labyrinth layer. Dilated blood vessels (arrows) are observed in *p600* ^−/−^ placenta. Scale bars indicate 50 µm. (C) Immunofluorescence staining of blood vessels in labyrinth areas with anti-laminin antibody. The blood vessels in labyrinth are dilated and sparse in *p600* KO animals at E12.5. Scale bars indicate 100 µm.

The formation of a proper placenta is critical for fetal development. In normal embryos at days from E8.5 to E10.5, branching morphogenesis of the labyrinth occurs to form dense villi, the site of exchange of nutrients. The development of placenta continues afterward and reaches maturity at E14.5. The mature placenta consists of three layers: the labyrinth, the spongio-trophoblast, and the maternal decidua [20]. Histological analysis of *p600* KO placenta showed that the labyrinth layer is thinner than that of the wild-type placenta (Figure 2A). Moreover, the labyrinth layer of the KO placenta contains abnormally dilated maternal blood vessels full of serum and with monocytes marginated along the endothelial inner lining of the blood vessels (Figure 2B).

During normal gestation, placental blood vessels play crucial roles by exchanging nutrients, oxygen, and wastes between embryo and mother [20]. In this perspective, we next examined the architecture of blood vessels in knockout animals by immunohistochemical staining with laminin antibody. Fluorescent immunohistochemistry showed that *p600* KO placenta contains dilated vessels with disorganized architecture in the labyrinth layer (Figure 2C). Taken together, we conclude that p600 is required for the formation of the placenta.

### *Sox2-Cre*-mediated *p600* Conditional Knockout Embryos Survive Longer than the Straight Knockout Embryos

**Figure 3:**
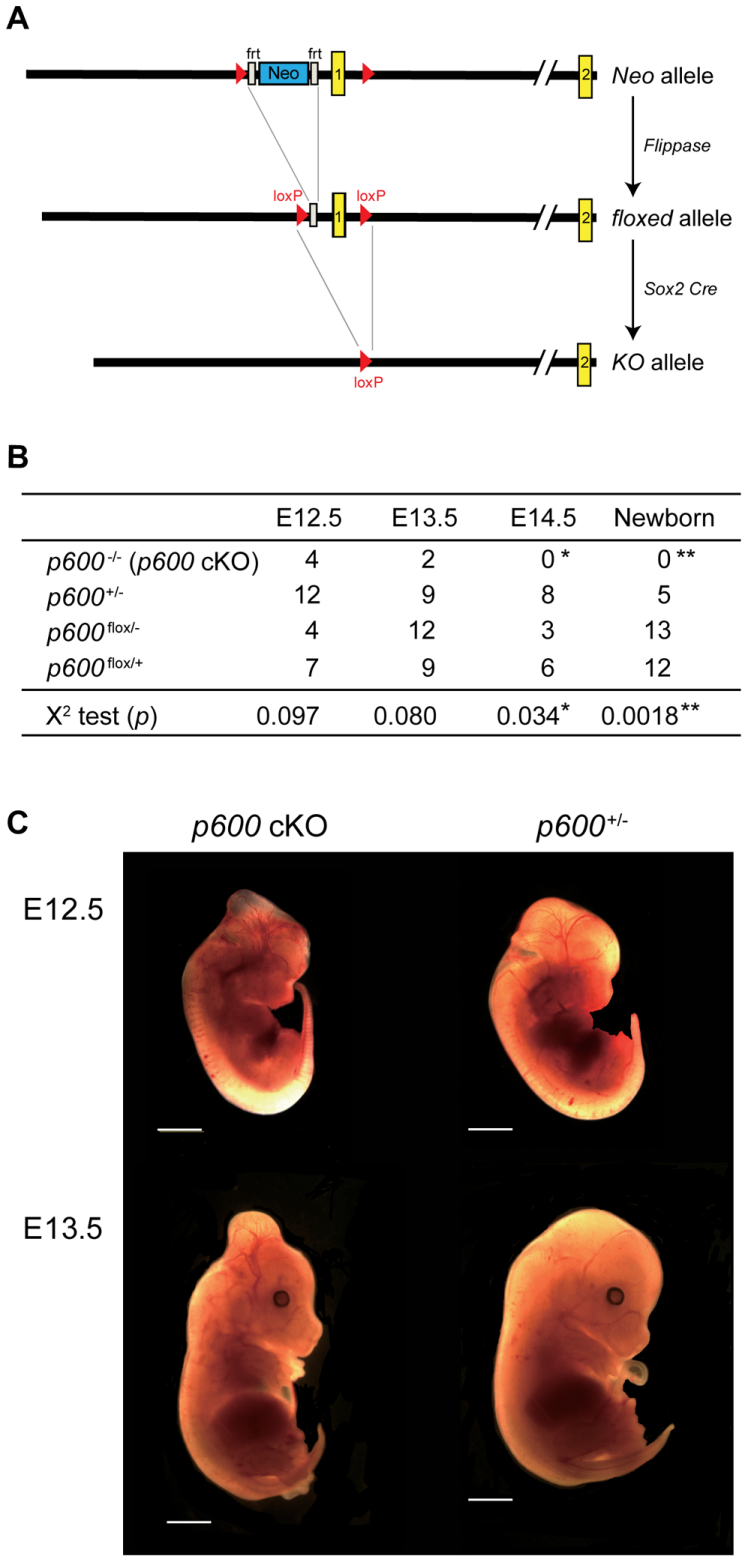
Conditional *p600* knockout specific to embryos. (A) Conditional deletion strategy of *p600* allele in embryos. The mice with ‘Neo allele’ were mated with transgenic mice which expresses *actin* promoter-driven *flippase*. This crossing eliminated the Neo cassette region between the frt sites (white rectangles), resulting in ‘floxed allele’. After crossing with Tg(*Sox2-Cre*) mice [21], the region between loxP sites (red triangles) including the first exon of *p600* is deleted where Cre recombinase is expressed, resulting in ‘KO allele’. (B) Genotype analysis of *Sox2-Cre*-mediated p600 knockout animals. Male mice of Tg (*Sox2-Cre*) *p600* ^+/−^ were crossed with female containing the homozygous *p600* floxed alleles (*p600* ^flox/flox^). Genotypes of resulting littermates were analyzed by PCR. The *p*-values of X^2^ test are shown. * and ** show significant differences between observed frequency of survival *p600* cKO embryos and expected frequency of 25%, according to Mendelian distribution of the genotypes with p-score <0.05 and <0.01, respectively. (C) Typical appearances of *p600* cKO and heterozygous *p600* ^+/−^ (*p600* ^+/−/^ *Sox2 Cre* ^+^) embryos at days E12.5 and E13.5. Scale bars indicate 1 mm.

These placental defects preclude to a clear definition of the embryonic roles of p600. To eliminate secondary influences of placental abnormalities we generated conditional knockout of *p600* using *Sox2* promoter-driven *Cre* transgene [Tg (*Sox2-Cre*)], which disrupts *p600* alleles in embryos but not in placenta [21]. We first produced *p600* floxed mutant mice, which possess loxP sites flanking the exon-encoding the initiating methionine, from the mice containing heterozygous ‘Neo allele’ (Figures 1A and 3A). To delete the Neo cassette inserted between frt (flippase recognition target) sites, the heterozygous mice were crossed with *ß-actin* promoter driven-flippase transgenic mice [22] (Figure 3A). Accordingly, we obtained mice containing the p600 ‘floxed allele’. The resulting mice were crossed with Tg (*Sox2-Cre*) mice [21] to delete the fragment containing the initiating methionine flanked by loxP sites in embryos (Figure 3A).

First, *p600* ^flox/+^ mice were consequently crossed with Tg (*Sox2-Cre*) mice to obtain *p600* ^+/−^ offspring with heterozygous *Sox2-Cre* allele (*p600* ^+/−/^ *Sox2-Cre*). Then to produce *p600* ^−/−^ conditional knockout (*p600* cKO) mice, male *p600* ^+/−/^ *Sox2-Cre* mice were crossed with female *p600* ^flox/flox^ mice. We analyzed the genotype and gross morphology of living embryos from E12.5 to E14.5 (Figures 3B and 3C, and S2). In the straight KO embryos generated by inter-crossing of *p600* ^+/−^ parents, only ∼4% of total living embryos were *p600* ^−/−^ at E12.5 and none of them were *p600* ^−/−^ at E13.5 (Figure 1B). In contrast, in the conditional knockout strain, 15% of total living embryos were *p600* ^−/−^ at E12.5 and 6% were *p600* ^−/−^ at E13.5 (Figure 3B). These results indicate that the survival period of *p600* ^−/−^ embryos is prolonged by the embryo-specific knockout. However, no living *p600* cKO embryos were observed after E14.5. While gross morphology of *p600* cKO embryos at E12.5 revealed mild growth retardation, the bulges sticking up from the middle of the head were observed in the mutant animals. This phenotype became more obvious at later stage such as E13.5 (Figure 3C), thereby suggesting an important role for p600 during brain development (see below and Discussion).

We next verified whether placental abnormalities found in the straight *p600* knockout are spared in the conditional knockout animals. As expected, no structural abnormalities in the labyrinth layer were observed in *p600* cKO animals (Figure S3A). Blood vessels of the labyrinth layer were unaffected in *p600* cKO animals as well (Figure S3B). Taken together, these data indicate that p600 is intrinsically crucial for the fetal development.

### Embryonic p600 is Essential for the Cardiac Development

**Figure 4:**
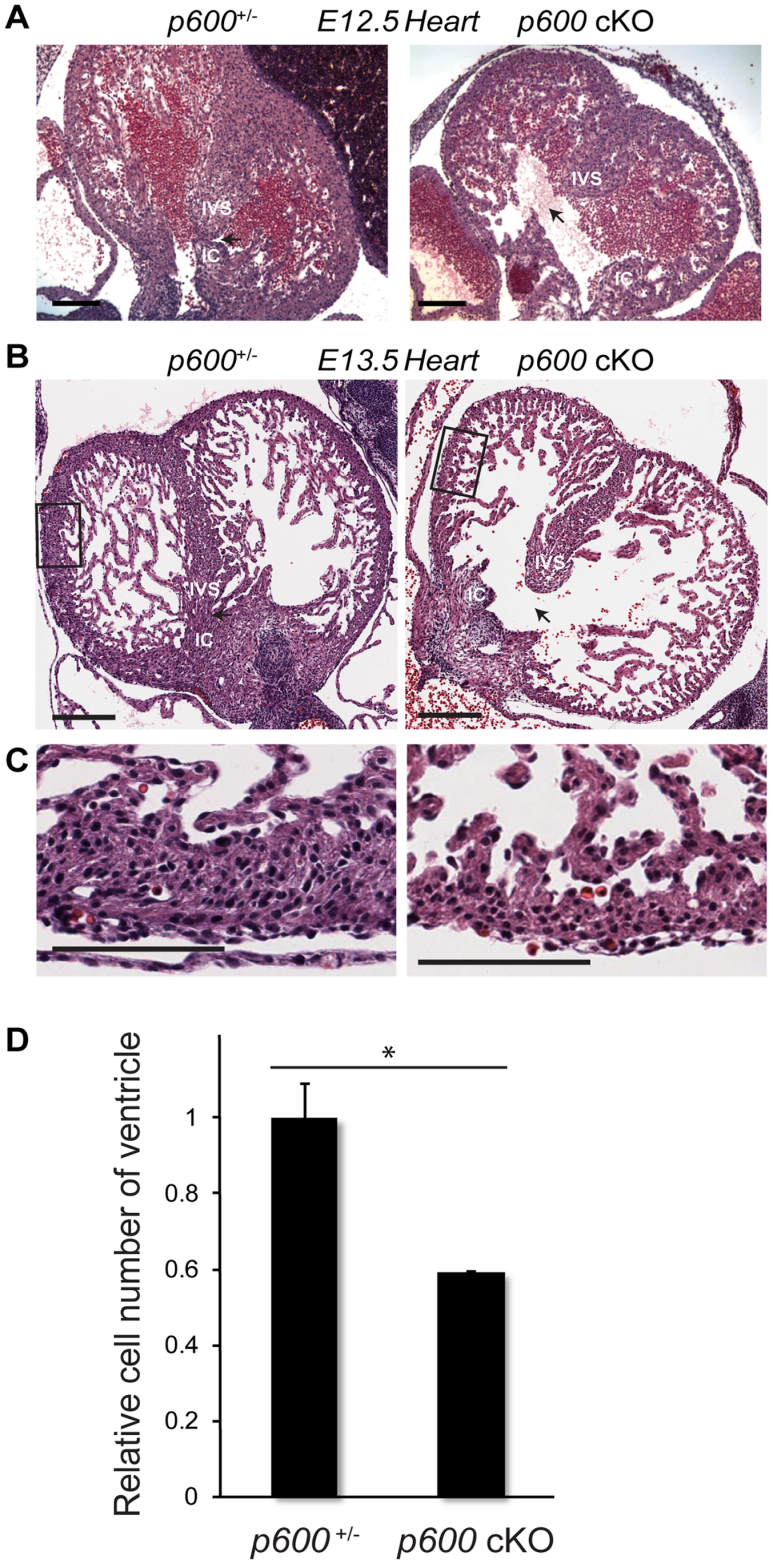
*Sox2-Cre*-mediated conditional knockout revealed that embryonic p600 is essential for heart development. (A) Impaired interventricular septum formation in *p600* cKO heart. From H&E stained transverse cross-sections of E12.5 embryos, the interventricular septum areas are shown. In the *p600* ^+/−^ (*p600* ^+/−/^ *Sox2-Cre* ^+^) heart (left), the interventricular septum (IVS) is fused with inferior atrioventricular endocardial cushions (IC), separating the left and right ventricles. On the other hand, *p600* cKO heart has developmental defects of the interventricular septum. Scale bars indicate 500 µm. (B) The *p600* ^+/−^ (left) and *p600* cKO (right) heart at E13.5. Scale bars indicate 200 µm. (C) Magnified images of ventricular wall. Magnified areas are indicated as boxes in (B). Scale bars indicate 100 µm. (D) Relative cell numbers of the ventricular wall in the *p600* cKO and *p600* ^+/−^ hearts at E13.5. Cell numbers of ventricular wall areas were estimated from the several serial sections and shown as relative values. The statistical significance of the difference was calculated with Student’s t-test. * shows *p*-value <0.05.

Histological analysis of *p600* cKO embryos revealed serious defects in cardiac development. In normal fetal heart, the interventricular septum developed from the inner surface of the ventricles is fused with the inferior atrioventricular endocardial cushions, separating left and right ventricles at E12.5 (Figure 4A, left) [23], [24]. In the cKO heart, development of the interventricular septum and the inferior atrioventricular endocardial cushions is immature, forming a large canal between left and right ventricles (Figure 4A, right). Although the volume of ventricles increased from E12.5, at E13.5, the formation of the ventricular septum remains immature in *p600* cKO (Figure 4B). Moreover, the cKO heart has a thinner ventricular wall when compared to the control heart (Figures 4C and 4D). From these observations, we conclude that loss of *p600* during embryogenesis is associated with defects in heart development. This may eventually lead to gross growth retardation and embryonic death.

**Figure 5:**
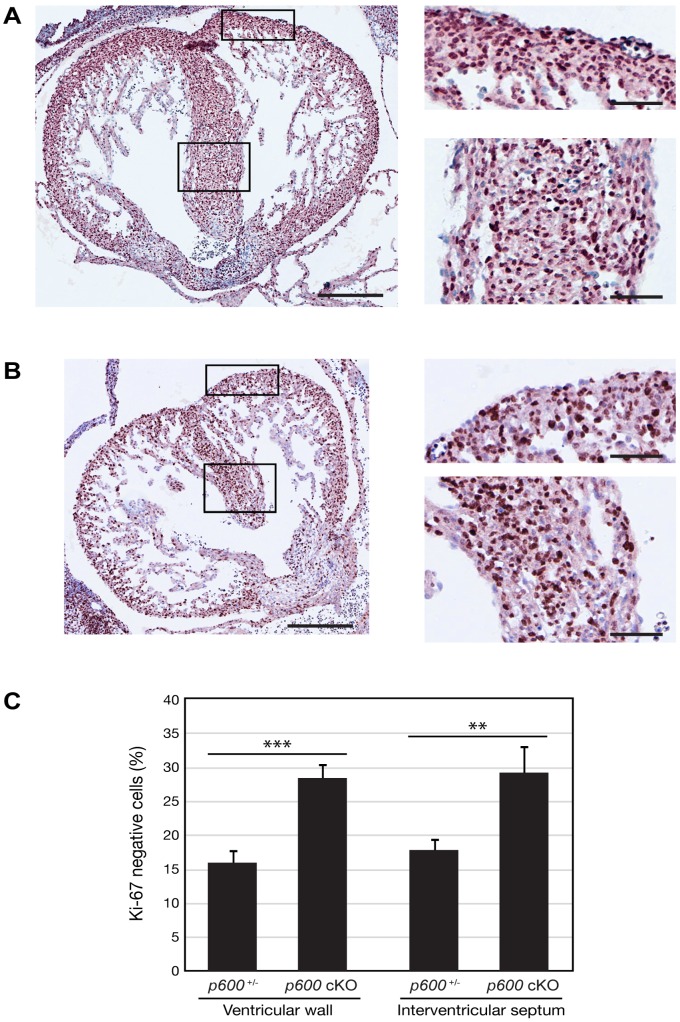
Proliferation defects in conditional *p600* knockout heart. Transverse sections of *p600* ^−/+^ (*p600* ^+/−/^ *Sox2-Cre* ^+^) (A) and *p600* cKO (B) heart at days E13.5 were stained with anti-Ki-67 antibody. After immunehistological staining, the samples were counterstained with hematoxylin. Magnified images of the ventricular wall (top) and the interventricular septum (bottom), the region indicated by rectangles, are shown in the right panels. Scales bars in the left and right panels indicate 500 and 200 µm, respectively. (C) The percentage of Ki-67 negative cells in the ventricular wall and the interventricular septum are shown. The *p*-values for Student’s t-test are shown in the graph. ** and *** show *p*-value <0.01 and <0.001, respectively.

To gain insights into the molecular mechanisms underlying developmental defects in the *p600* cKO heart we first analyzed proliferation of cardiomyocytes by immunohistochemistry with anti-Ki-67 antibody (Figures 5). Ki-67, a cellular marker for proliferation, is expressed during all cell cycle stages in proliferating cells but not in quiescent or terminally differentiated cells [25]. The ratio of Ki-67-negative cells to the total cells was significantly increased in the cKO heart when compared to that in the control heart (Figure 5C). This result suggests abnormal proliferation of cardiac cells in *p600* cKO embryos.

We have attempted to detect apoptotic cells by TUNEL assay as well as immunohistochemistry with the antibody specific to the cleaved caspase-3. Interestingly, apoptotic cells were barely detected in the cKO and control heart (data not shown). These data are consistent with the H&E data, showing that there is no detectable dead cardiomyocytes in the cKO and control heart (Figure 4). Taken together, these data indicate that developmental defects in the *p600* cKO heart could be due to deceleration of cell proliferation rather than acceleration of cell death.

**Figure 6:**
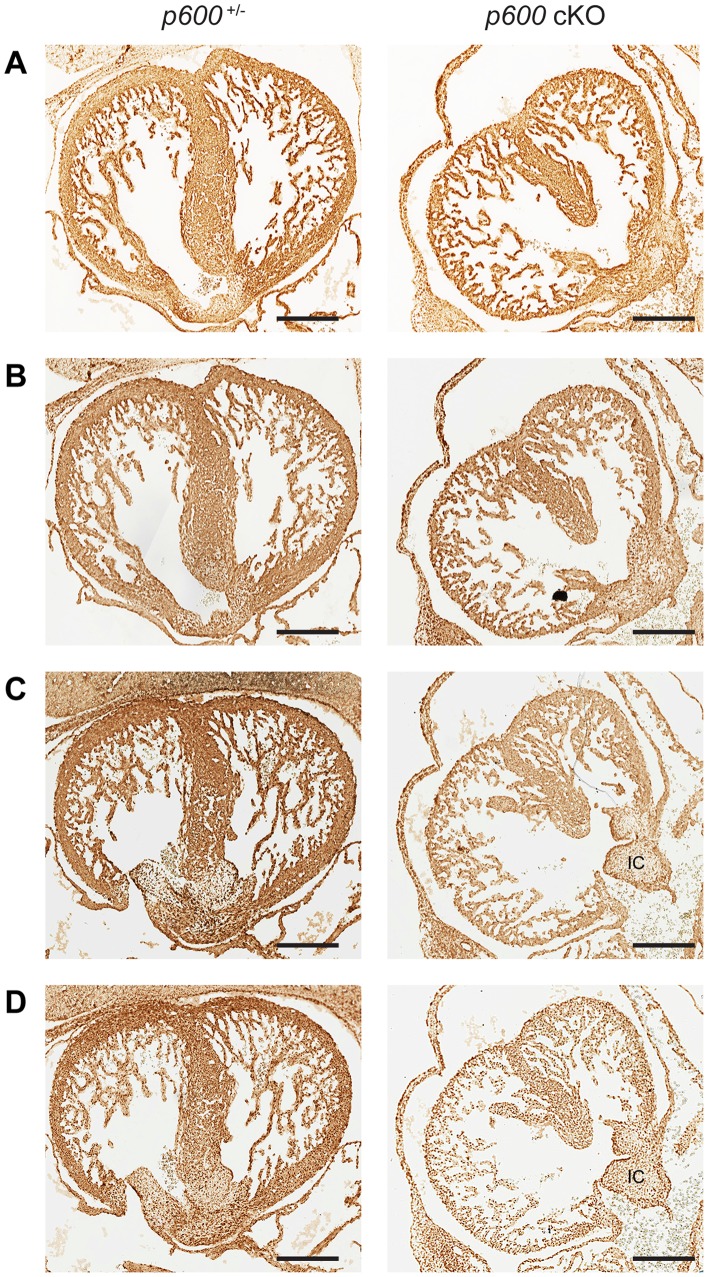
Defects in FAK activation in conditional *p600* knockout heart. Transverse sections of *p600* ^−/+^ (*p600* ^+/−/^ *Sox2-Cre* ^+^) (left) and *p600* cKO (right) heart at days E13.5 were stained with anti-desmin (A), anti-FAK (B), anti-phospho-FAK (Tyr397) (C), and anti-MEF2 (D) antibodies. In panels C and D, the position of the inferior atrioventricular endocardial cushions (IC) is labeled. Scale bars indicate 200 µm.

To examine whether cardiomyocytes differentiate properly we immunostained the sections with antibody against desmin, a subunit of intermediate filaments specific to muscle tissues. The stained images indicate that the cKO heart express desmin at the level comparable to the control heart (Figure 6A).

FAK is a non-receptor protein tyrosine kinase involved in integrin-mediated signal transduction. FAK is activated by the establishment of integrin-extracellular matrix interactions and growth hormones. Activation of FAK results in autophosphorylation at Tyr397, serving as a binding site for the Src homology 2 (SH2) domains of Src-family tyrosine kinases and the p85 subunit of phosphatidylinositol 3-kinase (PI3K) [26]. By knocking down p600 expression with shRNA, we previously demonstrated that p600 is involved in activation of FAK in cultured fibroblasts [1]. Moreover, conditional knockout of *FAK* in the embryonic heart has been shown to cause the similar heart defects, i.e., ventricular septal defects and thin ventricular walls [27]. Accordingly, we examined FAK activation in the *p600* cKO heart. No obvious difference was observed in FAK expression between the cKO and control heart (Figure 6B). In contrast, the overall level of phosphorylated FAK at Tyr397 in the cKO heart was significantly lower than that in the control heart (Figure 6C). Taken together, these results indicate defective FAK activation in the heart of cKO embryos.

We next determined the levels of myocyte enhancer factor 2 (MEF2), which is under regulation of FAK [27] (Figure 6D). Like phosphorylated FAK, expression of MEF2 was also reduced in the cKO heart. Interestingly, both phosphorylated FAK and MEF2 accumulated at the surface cells of the inferior atrioventricular endocardial cushions in the cKO heart (Figures 6C and 6D). Taken together, we conclude that p600 may play a role in the activation of FAK signaling pathways during cardiac development.

### Embryonic p600 is Required for Development of the Liver

**Figure 7:**
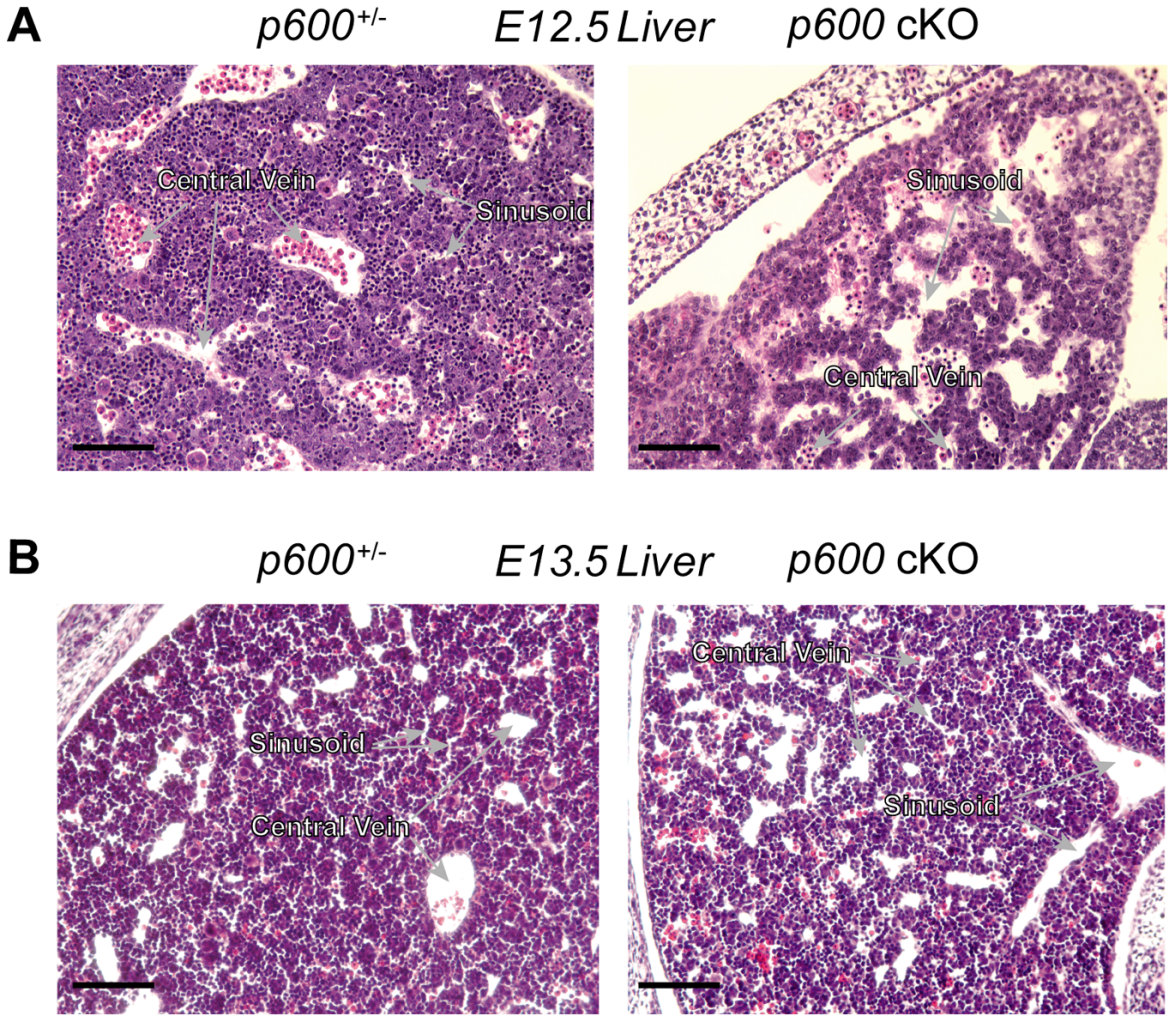
Embryonic p600 is required for liver development. H&E stained transverse sections of *p600* ^−/+^ (*p600* ^+/−/^ *Sox2-Cre* ^+^) (left) and *p600* cKO (right) liver at days E12.5 (A) and E13.5 (B). Note that *p600* cKO livers have less densely packed parenchymal hepatocytes. Scale bars indicate 100 µm.

In addition to the cardiac defects, pleiotropic defects were observed in *p600* cKO embryos. At E12.5, cKO liver displays reduced cellularity and increased empty space (or the hepatic sinusoids) (Figure 7A, right), indicative of less dense packing of parenchymal hepatocytes, when compared to the control liver (Figure 7A, left). At E13.5, in the control liver, parenchymal hepatocytes become denser and form the structural units called hepatic lobules (Figure 7B, left). On the other hand, blood vessels including sinusoids are very dilated in the mutant liver (Figure 7B, right). These results indicate that p600 plays an essential role in liver development.

### Embryonic p600 is Required for Development of the Brain

**Figure 8:**
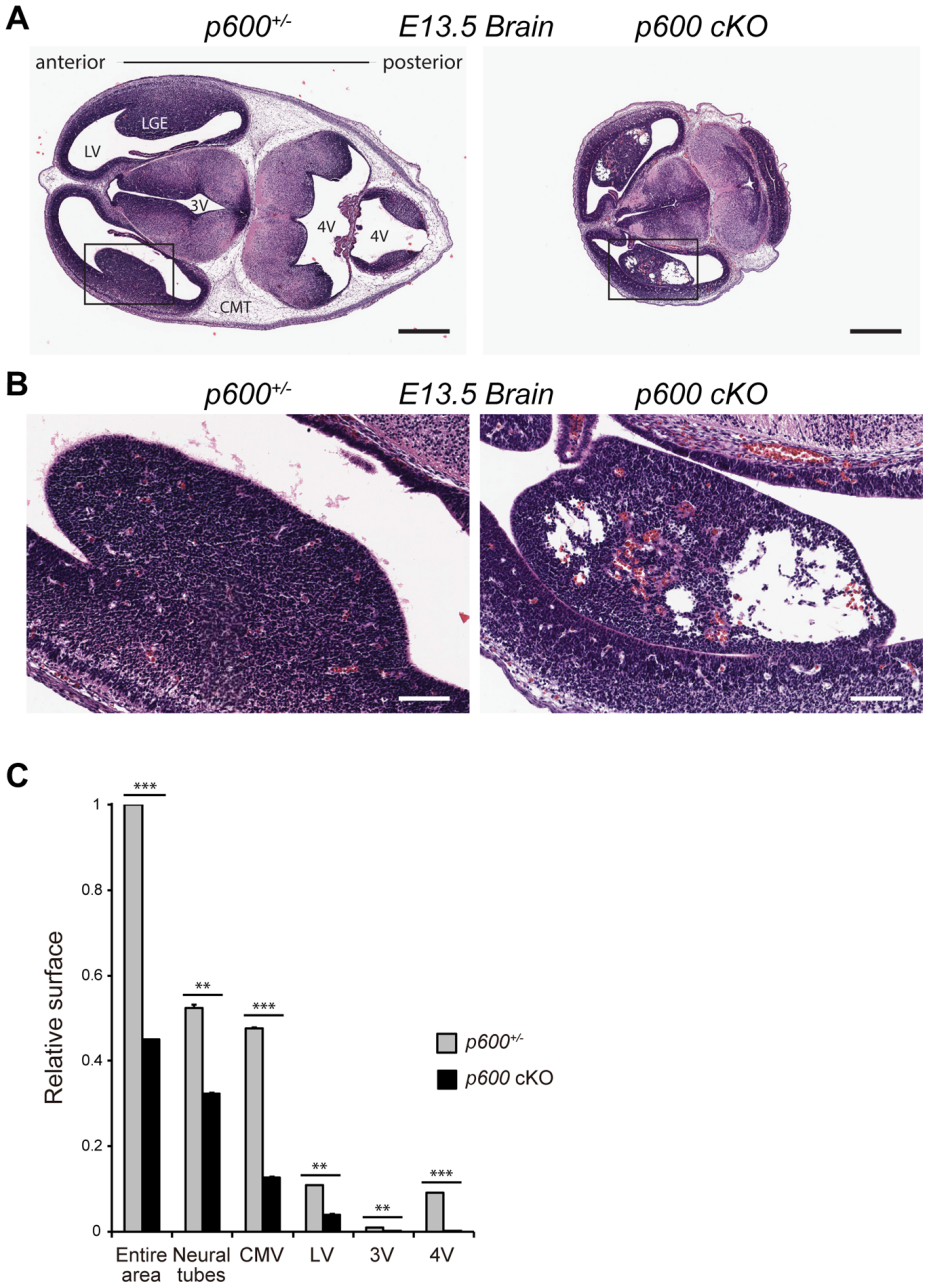
Embryonic *p600* is required for brain development. (A) Embryonic p600 is required for brain development. H&E stained transverse sections of *p600* ^−/+^ (left) and *p600* cKO (right) brain at E13.5. Note that the cKO embryo has considerably smaller brain with markedly small ventricular spaces and cephalic mesenchyme tissues. Abbreviations: LGE, lateral ganglionic eminence; CMT, cephalic mesenchyme tissues LV, lateral ventricle; 3V, third ventricle; and 4V, fourth ventricle. Scale bars indicate 500 µm. (B) Magnified images of the lateral ganglionic eminence area of *p600* ^−/+^ (*p600* ^+/−/^ *Sox2-Cre* ^+^) (left) and *p600* cKO (right) brain, the region indicated by rectangles in (A). Scale bars indicate 100 µm. (C) The relative surface areas measured from the sections shown in (A) and the adjacent sections. The statistical significance of the differences was calculated with Student’s t-test. ** and *** show *p*-value <0.01 and <0.001, respectively.

Consistent with the abnormal gross morphology of the head observed in *p600* cKO embryos (Figure 3C), analysis of brain serial cross sections revealed significant defects. p600 cKO embryos have considerably small brain when compared to the control littermates (Figure 8). Furthermore, cKO brain appears to be compressed especially in the posterior portion. Interestingly, although there is no significant difference in the area of the neural tubes between the cKO and control brain, the area of ventricles and cephalic mesenchyme tissues is significantly reduced in the cKO brain (Figure 8C). Especially, the fourth ventricle is mostly closed through all cross sections. Moreover, severe cell death with cavitation was observed in the lateral (Figure 8B) and medial (data not shown) ganglionic eminences, which localize below the cortex of forebrain. Thus, p600 is important for brain development.

## Discussion

In summary, we demonstrated that p600 plays essential roles in embryonic development and formation of placenta. Moreover, by generating conditional knockout mice in which *p600* is deleted selectively in embryos but not in placenta, we showed that embryonic p600 is critical for the development of fetal organs. The embryonic lethal phonotype was totally unexpected for us since *push*, the *Drosophila* counterpart to *p600*, is not essential for development to adult flies [13], [28]. Three independent *push* mutants have been characterized; two ethyl methanesulfonate-induced mutants, which possess nonsense mutations at the N-terminal region of push and a transposon-induced mutant, which has the P-element insertion at just upstream of the translation start site of *push* [28]. Importantly, *Drosophila* embryos harboring these *push* mutations develop into adult flies and are viable despite abnormalities in behavior and synaptic transmission at the neuromuscular junction [13], [28], [29]. Mouse p600 and *Drosophila* push have similar protein sizes (5,180 and 5,322 amino acids, respectively) and share significant sequence similarities in the broad regions, which cover >95% of the entire proteins. Nevertheless, our data demonstrate that p600 plays specific and essential roles during development in mammal.

First, we demonstrated that conditional knockout of *p600* in the epiblast cells causes embryonic lethal phenotype associated with cardiac developmental problems, including ventricular septal defects and thin ventricular walls (Figure 4). Immunohistological analysis indicates that the *p600* knockout cardiomyocytes have defects in activation of FAK and expression of MEF2, which is regulated by FAK [27] (Figure 6). Importantly, MEF2 has been shown to play a crucial role in myogenesis and cardiac development [30]. Consistent with our results, conditional knockout of FAK with Cre element under the control of the myosin light chain 2a promoter (MLC2a-Cre), which deletes FAK specifically in embryonic heart, results in ventricular septal defects and thin ventricular walls. Thus, failure in FAK activation could be one of the mechanisms underlying cardiac developmental defects in *p600* cKO.

In support of our findings, the relevance of p600 to congenital heart defects (CHD), which are structural problems with the heart present at birth, has been suggested [31]. Ventricular septal defect (VSD), atrial septal defects (ASD), and tetralogy of Fallot (TOF) are known to be the most common CHD. To identify genes responsible for CHD, RNA arbitrarily primed-PCR fingerprinting was performed using RNAs isolated from three types of CHD and non-CHD hearts. From these screenings, p600 (hCalo) was identified as a highly expressed gene in the VSD samples, suggesting a role of p600 in the ventricular septum formation in human development.

As p600 is important for proliferation cardiomyocytes (Figure 5), it could be also critical for the proliferation of neural progenitors in the developing brain. In addition, suppression of p600 expression in the fetal brain by *in utero* electroporation of p600 RNA interference revealed a key role for p600 in neuronal migration in the developing neocortex [7]. Thus, defects in neurogenesis and neural migration could be among the causes underlying the microcephalic phenotype of *p600* cKO embryos. At this stage, we cannot exclude the possibility that apoptosis, autophagy and/or necrosis may also contribute to the brain phenotype. Further in-depth studies are underway to unravel the multiple roles of p600 in the developing brain.

In the course of this work, the Kwon lab has independently developed *UBR4/p600* knockout mice that, like our mice, also show an embryonic lethal phenotype [32]. However, their knockout animals display developmental defects at significantly earlier stage (E9.0) by pleiotropic abnormalities, including defects in the yolk sac, precluding a clear analysis of organ development at later stages. Although it is uncertain why death occurred at different stages in the two independent knockouts, a possibility would reside in the different knockout strategies employed. While we deleted a *p600* region containing the first exon (Figure 1A), Tasaki et al. replaced a region containing exons 36 through 42, which encodes the UBR box, with a cassette encoding the internal ribosome entry site (IRES), tau-lacZ, and poly-A so that the lacZ reporter is expressed from the *p600* transcription start site [32]. In their strategy, the p600 N-terminal region (exons 1 through 35) and lacZ is transcribed as a bicistronic mRNA. Accordingly, it would be possible that the truncated p600 protein, which is potentially expressed in the knockout animals, has inhibitory functions in the fetal development. In sum, our work with the embryo-specific knockout animals presents the great and unique advantage to understand roles of p600 in fetal development. Further studies will address the molecular mechanisms whereby p600 contributes to fetal development.

## Supporting Information

Figure S1 **Genotype and protein analyses of** ***p600*** **straight knockout embryos.** (A) Genotype analysis of the knockout embryos by Southern blotting. Genomic DNAs isolated from embryos were digested with BamHI and SpeI and hybridized with the ^32^P-labeled probe (Figure 1A). The probe hybridizes with 2.5 and 4.3 kbp fragments in *WT* and *KO* alleles, respectively. The identified genotypes of *p600* are indicated on the top. The positions of DNA molecular weight markers are indicated on the left. (B) Genotype analysis of the knockout embryos by PCR. Genotypes of p600 were determined by genomic PCR using primer sets, which amplify *WT* (top) and *KO* alleles (bottom) (see Materials and Methods and Figure 1A). (C) Detection of p600 protein in the knockout embryos. Crude protein extracts were prepared from a day E10.5 embryo and performed Western blotting with anti-p600 (top) and anti-actin (bottom) antibodies. The genotypes of embryos are shown on the top. The positions of protein molecular weight markers are indicated on the right.
(TIF)

Figure S2 **Genotyping of** ***p600*** **conditional knockout embryos.** The genotypes of *p600* cKO embryos were determined by genomic PCR with primer sets which amplify *WT and floxed* alleles (top), *KO* allele (middle), and *Sox2-Cre* transgene as described in Material and Methods. Determined genotypes are indicated on the top.
(TIF)

Figure S3 **Structural abnormalities of placentas found in the straight** ***p600*** **KO are recovered in** ***p600*** **cKO animals.** (A) H&E staining sections of days E12.5 and E13.5 placentas isolated from *p600* cKO and *p600^−/+^* (*p600* ^+/−/^ *Sox2-Cre*) animals. Scale bars indicate 500 µm (top) and 50 µm (bottom). The labyrinth layers are indicated by allow lines. (B) Immunofluorescence staining of blood vessels in labyrinth areas with anti-laminin antibody. There results indicate that there is no significant difference between labyrinths isolated from *p600* cKO and *p600^−/+^* animals. Scale bars indicate 100 µm.
(TIF)
