# Supplementary figures and images for: p600 Plays Essential Roles in Fetal Development
Source: PLoS One. 2013 Jun 18;8(6):e66269. doi: 10.1371/journal.pone.0066269 (PMC3688873; doi:10.1371/journal.pone.0066269)

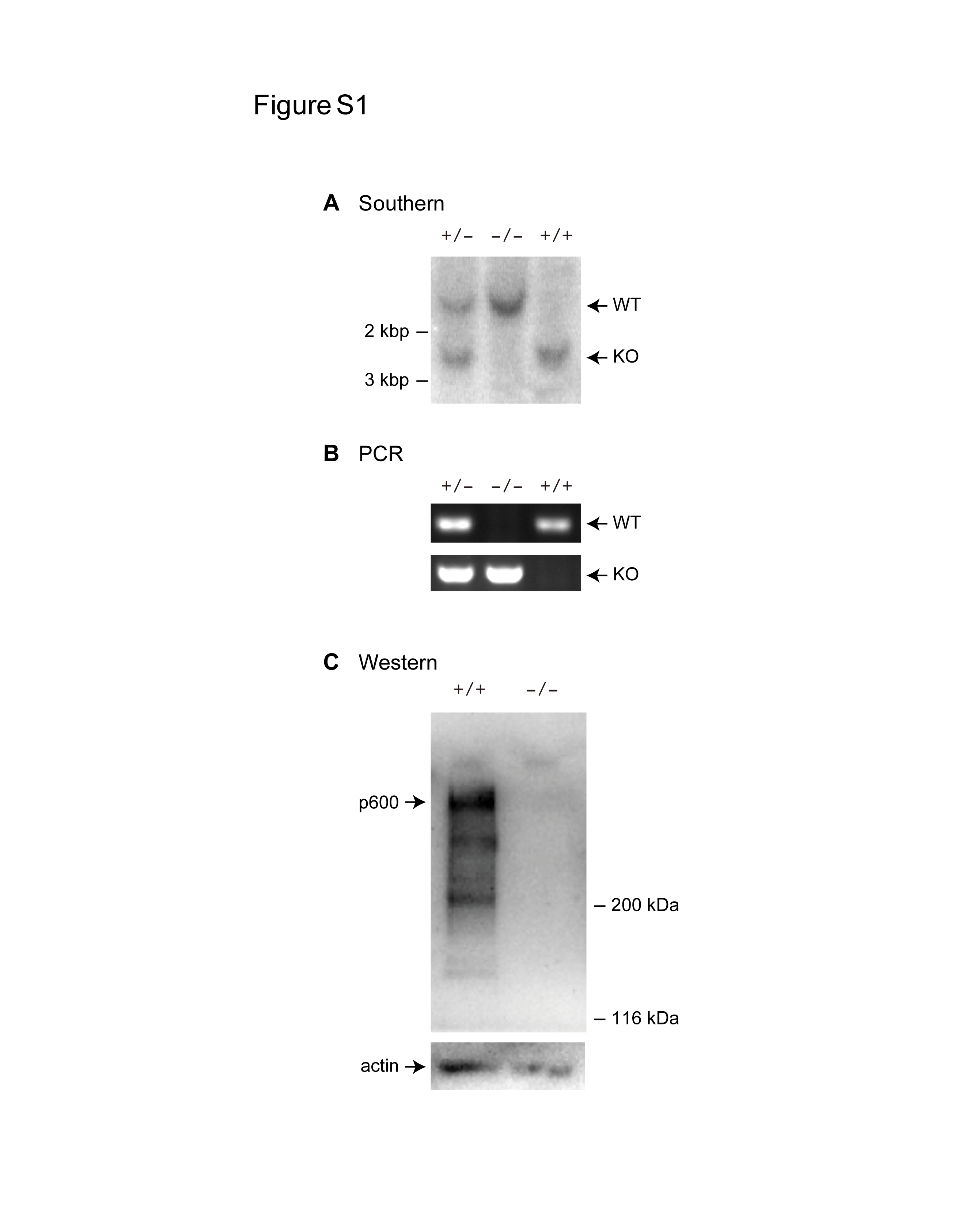

Supplement: Figure S1 — Genotype and protein analyses of p600 straight knockout embryos. (A) Genotype analysis of the knockout embryos by Southern blotting. Genomic DNAs isolated from embryos were digested with BamHI and SpeI and hybridized with the 32P-labeled probe (Figure 1A). The probe hybridizes with 2.5 and 4.3 kbp fragments in WT and KO alleles, respectively. The identified genotypes of p600 are indicated on the top. The positions of DNA molecular weight markers are indicated on the left. (B) Genotype analysis of the knockout embryos by PCR. Genotypes of p600 were determined by genomic PCR using primer sets, which amplify WT (top) and KO alleles (bottom) (see Materials and Methods and Figure 1A). (C) Detection of p600 protein in the knockout embryos. Crude protein extracts were prepared from a day E10.5 embryo and performed Western blotting with anti-p600 (top) and anti-actin (bottom) antibodies. The genotypes of embryos are shown on the top. The positions of protein molecular weight markers are indicated on the right. (TIF) [file pone.0066269.s001.tif]

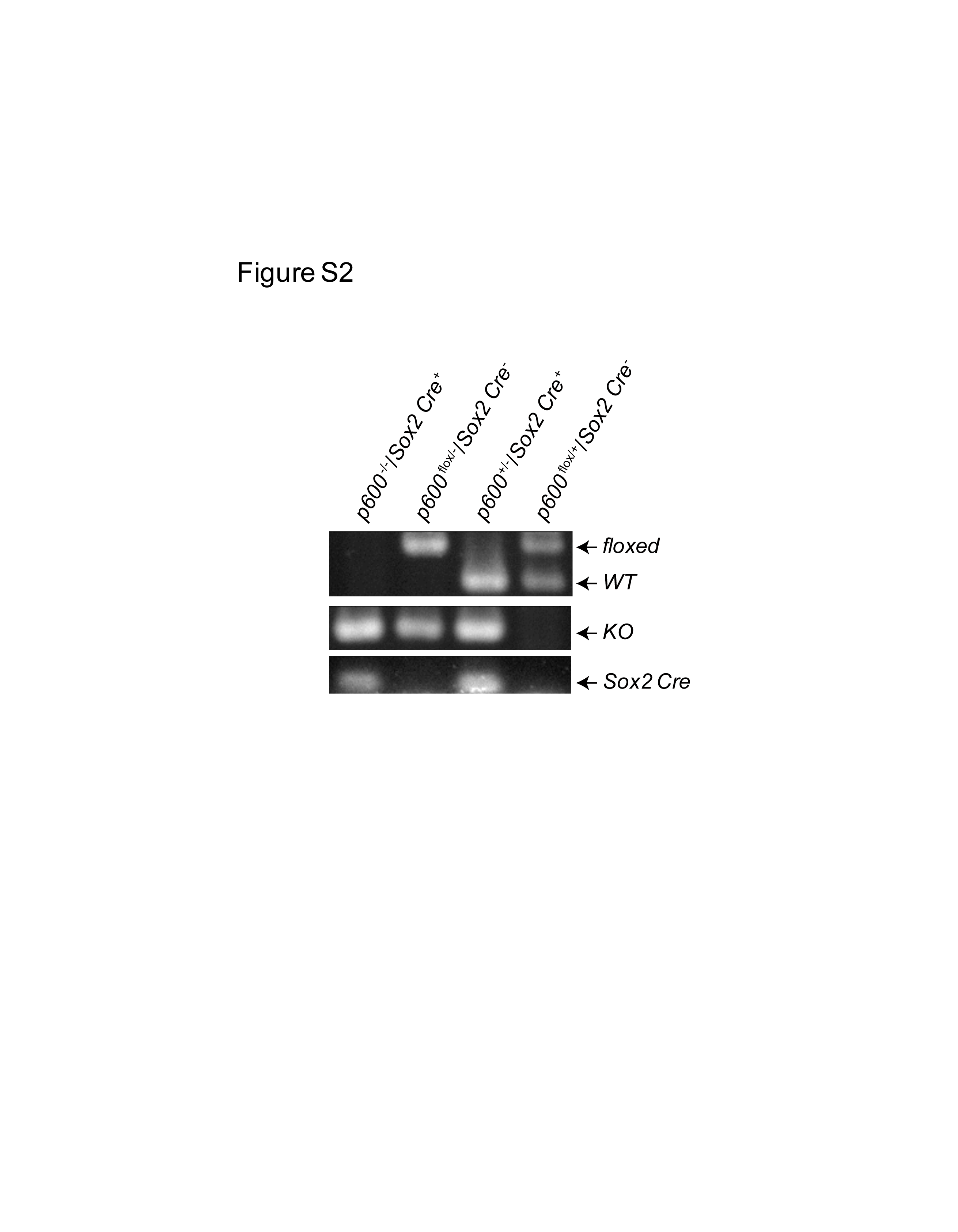

Supplement: Figure S2 — Genotyping of p600 conditional knockout embryos. The genotypes of p600 cKO embryos were determined by genomic PCR with primer sets which amplify WT and floxed alleles (top), KO allele (middle), and Sox2-Cre transgene as described in Material and Methods. Determined genotypes are indicated on the top. (TIF) [file pone.0066269.s002.tif]

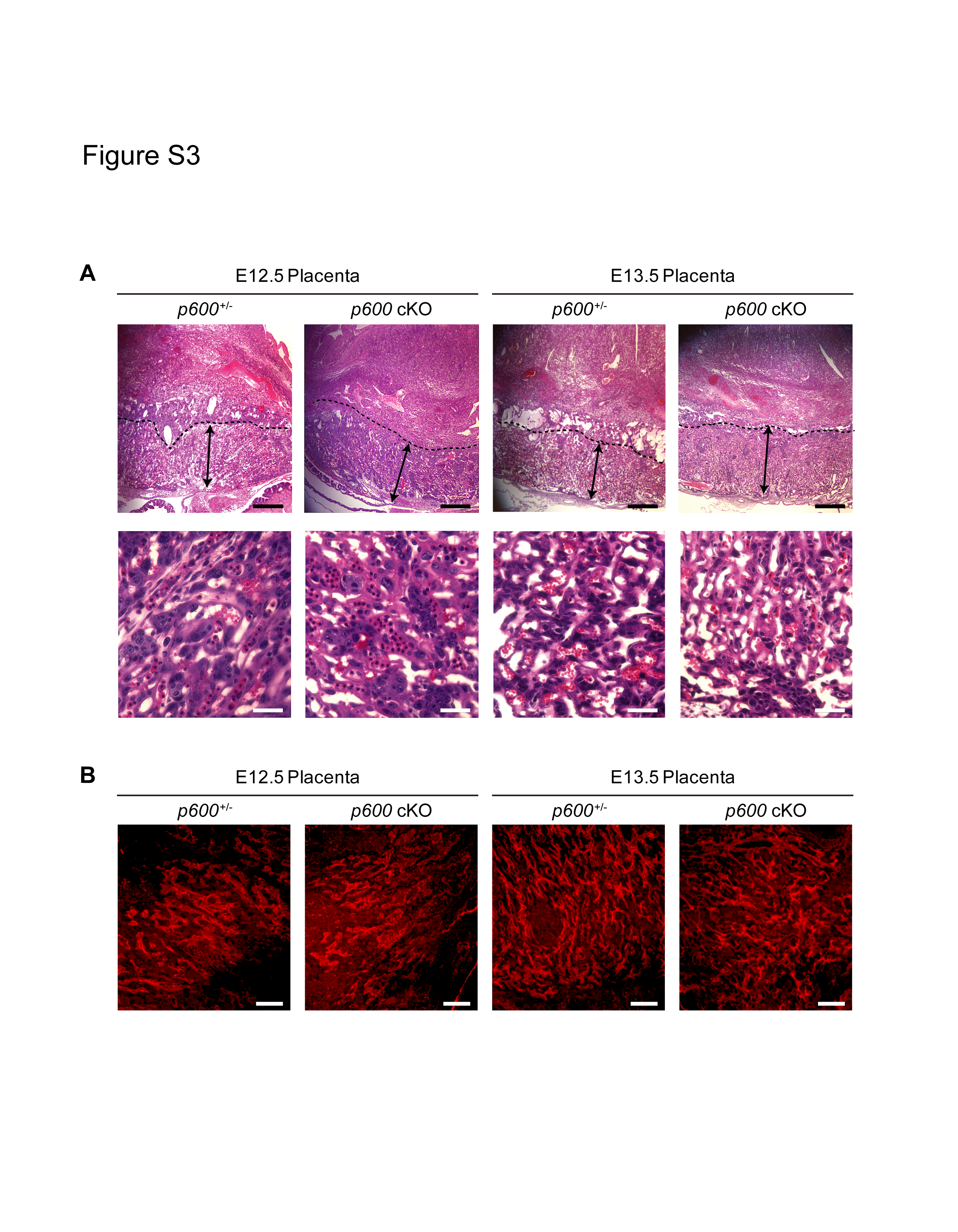

Supplement: Figure S3 — Structural abnormalities of placentas found in the straight p600 KO are recovered in p600 cKO animals. (A) H&E staining sections of days E12.5 and E13.5 placentas isolated from p600 cKO and p600−/+ (p600 +/−/ Sox2-Cre) animals. Scale bars indicate 500 µm (top) and 50 µm (bottom). The labyrinth layers are indicated by allow lines. (B) Immunofluorescence staining of blood vessels in labyrinth areas with anti-laminin antibody. There results indicate that there is no significant difference between labyrinths isolated from p600 cKO and p600−/+ animals. Scale bars indicate 100 µm. (TIF) [file pone.0066269.s003.tif]
